# The Otago Exercise Program compared to falls prevention education in Zuni elders: a randomized controlled trial

**DOI:** 10.1186/s12877-022-03335-6

**Published:** 2022-08-09

**Authors:** Debra L. Waters, Janet Popp, Carla Herman, Donica Ghahate, Jeanette Bobelu, Vernon S. Pankratz, Vallabh O. Shah

**Affiliations:** 1 Department of Internal Medicine, School of Medicine, University of New Mexico, Albuquerque, NM 87131 USA; 2 Department of Medicine and School of Physiotherapy, University of Otago, Dunedin, 9054 New Zealand; 3 Division of Physical Therapy, School of Medicine, University of New Mexico, Albuquerque, NM 87131 USA; 4 Department of Internal Medicine and Biochemistry, School of Medicine, University of New Mexico, Albuquerque, NM 87131 USA

**Keywords:** American Indians, Fall prevention, Older adults, Strength and balance

## Abstract

**Background:**

When a Zuni elder sustains a fall-related injury, the closest tribal skilled nursing facility is 100 miles from the Pueblo and no physical therapy services are available. Thus, fall prevention strategies as a primary intervention to avert injurious falls and preserve aging in place are needed. The objective of the study is to compare the effectiveness of a community health representative (CHR)-delivered, culturally-adapted Otago Exercise Program (OEP) fall prevention program compared to the standard of care education-based fall risk management.

**Methods:**

“Standing Strong in Tribal Communities: Assessing Elder Falls Disparity” is mixed-methods research with a randomized controlled trial. The CHRs will be trained to deliver the culturally-adapted OEP trial and offer advantages of speaking “Shiwi” (Zuni tribal language) and understanding Zuni traditions, family structures, and elders’ preferences for receiving health information. Focus groups will be conducted to assure all materials are culturally appropriate, and adapted. A physical therapist will train CHRs to screen elders for falls risk and to deliver the OEP to the intervention group and education to the control group. Up to 400 Zuni elders will be screened by the CHRs for falls risk and 200 elders will be enrolled into the study (1:1 random allocation by household). The intervention is 6 months with measurements at baseline, 3, 6 and 12 months. The primary outcome is improved strength and balance (timed up and go, sit to stand and 4 stage balance test), secondary outcomes include falls incidence, self-efficacy using Attitudes to Falls-Related Interventions Scale, Medical Outcomes Study Short Form 12 (SF-12v2) and Self-Efficacy for Managing Daily Activities.

**Discussion:**

Fall prevention for Zuni elders was identified as a tribal priority and this trial is built upon longstanding collaborations between the investigative team, Zuni tribal leaders, and multiple tribal health programs. Delivery by the CHRs make this model more acceptable, and thus, more sustainable long term. This study has the potential to change best practice for elder care in tribal and rural areas with limited access to physical therapist-delivered fall prevention interventions and aligns with tribal goals to avert fall-related injury, reduce healthcare disparity, and preserve elder’s independence.

**Trial registration:**

NCT04876729

## Background

A growing source of health disparities is the epidemic of falls and associated injuries in socio-economically disadvantaged and geographically isolated American Indian communities. Falls are particularly serious for older American Indians who experience more comorbidity and chronic illnesses than the general population [1]. The unintentional fall death rate is higher in American Indians from the Southwestern US than other regions of the US and is higher in New Mexico (NM) than other regions of the US [2]. The latest available data in 2017, reported that unintentional fall-related injury was the leading cause of unintentional injury-related death, hospitalization and emergency department visits for older New Mexicans [3]. A Tribal Behavioral Risk Factor Surveillance Survey of NM tribes conducted by the Albuquerque Area Southwest Tribal Epidemiology Center reported that 32% of American Indian adults 65 + experienced at least one fall in the past three months. Among those who reported a fall, 30% sustained an injury [4]. Compounding these statistics, the fall-related mortality rates increased dramatically from age 50 years, which is 15 years younger than the national trend [5]. Fall-related injuries place older adults at risk of losing independence, and the New Mexico Department of Health reports that only 25% of adults hospitalized for a fall-related injury are discharged directly home [5]. The Zuni Pueblo in New Mexico is geographically isolated with limited access to rehabilitative services. If a Zuni elder sustains a fall-related injury, the closest tribal assisted living or skilled nursing facility is 100 miles from the reservation. Thus, fall risk and rates reduction is essential for this vulnerable community.

Many falls prevention interventions focus on improving lower body function through strength, gait, and balance training, in addition to the reduction of certain medications, removing home hazards, and correcting vision impairments [6–11]. However, there is very little data on strength, balance, and lower body function in older American Indians. The only study that objectively measured lower body functioning using the short physical performance battery test (SPPB) in a South-eastern tribe of elders reported that 51.8% of the sample had a baseline score of 9 or less (mean 8.8 ± 3.3) indicating poor physical function. The score worsened with age and was negatively associated with physical inactivity, vision loss and medical comorbidities [12]. Therefore, research on adapting existing evidence-based programs to American Indians, and testing their effectiveness, is still lacking.

In this mixed-methods research with a randomized controlled trial, we will culturally tailor the evidence-based falls prevention program, Otago Exercise Program (OEP) [13–17], and Centre for Disease Control (CDC) STEADI patient education tools [18] to evaluate their effectiveness in reducing fall risk factors and preventing falls in Zuni elders. To address elder falls disparity, we will train Zuni Community Health Representatives (CHRs), rather than Physical Therapists to deliver the adapted OEP. The CHRs are paraprofessional, indigenous, community-based, and trained healthcare staff who make excellent health advocates. They speak the Zuni tribal language (“Shiwi”) and understand family structures, traditions of the Zuni people, and elders’ preferences for receiving health information. The research objective is to compare the effectiveness of an adapted OEP that is CHR-delivered compared to the standard of care, which is an education-based fall risk management approach.

Our study was designed to accomplish the following specific aims:

1. To foster a sustainable multi-directional, participatory collaboration between the Zuni’s tribal leadership, stakeholders, Zuni Indian Health Services, and University of New Mexico Health Sciences Centre to enhance fall prevention training, education, and research.
2. To culturally adapt the evidence-based OEP for use with the Zuni elder population, by training CHRs to utilize the CDC STEADI toolkit for fall risk screening and the education control group.
3. To compare the effectiveness of the adapted OEP to an education-based fall risk management program in improving strength and balance (decreasing falls risk) and reducing falls among Zuni elders.
4. To compare the effectiveness of the adapted OEP to an education-based fall risk management program in improving overall health status, self-management of daily activities, and social engagement. 

Our proposed work is innovative by building on our collaboration with the CHRs and tribal leadership. In partnership, we will culturally adapt the OEP for use in an American Indian community, test a model of expanding the role of trained CHRs in preventing falls using OEP in people’s homes, and develop a model for fall risk reduction to expand the role of Medicaid-reimbursed CHRs.

## Methods

### Design and setting

**Fig. 1:**
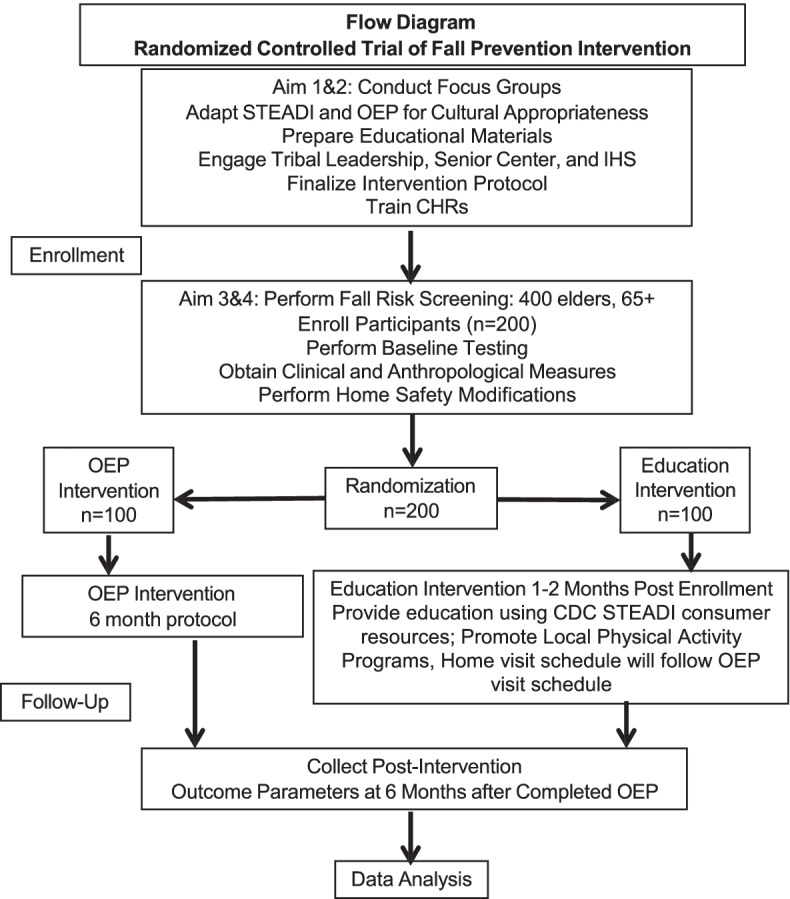
Flow diagram randomized controlled trial of fall prevention Iintervention

Our overarching aim of this study is to reduce falls risk health disparity among Zuni elders and to develop research capacity and resources for the Zuni Pueblo and it’s residents. It is a mixed-methods approach with a randomized controlled trial set on the Zuni Pueblo in New Mexico and the study flow chart is depicted in Fig. 1.

### Participant characteristics

#### Inclusion

Zuni elders will be eligible to participate if they are Zuni tribal members aged 65 years and older who demonstrate elevated fall risk according to 2 or more of the following outcomes: (1) Timed Up and Go ≥ 12 s; (2) 30 Second Chair Stand Test below age and sex norms; (3) Inability to complete the Four Stage Berg Balance Test; (4) Four or more positive responses on the *CDC Stay Independent: Check Your Risk for Falling*; or (5) history of 2 or more falls during past year or one injurious fall during past year.

#### Exclusion

The exclusion criteria include: (1) Self-reported diagnosis of terminal cancer in the last 6 months; (2) Currently on dialysis; (3) Mini-Cog score of 0 to 1; (4) Unwillingness to consent to participate; (5) Unable to walk with or without an assistive device; or (6) Legally blind. Potential participants will be screened and those eligible will be randomized into CHR-led OEP arm or an educational intervention control arm.

#### Specific aims

Aim 1 is to foster a sustainable multi-directional, participatory collaboration between the Zuni’s tribal leadership, stakeholders, Zuni Indian Health Services, and the University of New Mexico Health Sciences Centre to enhance community engagement in fall prevention training, education, and research. This will be achieved through a relationship with a Tribal Advisory Panel (TAP) that was convened and met twice to review the proposed study protocol and provided feedback and guidance to the investigators regarding conduct of the study. Liaisons from existing tribal health programs have been identified and have agreed to participate as TAP members. Community coordinators are part of the health programs at the Zuni Pueblo and will attend the quarterly TAP. The researchers based at the University of New Mexico will also attend quarterly meetings with the TAP, and discussions will focus on CHR activities and project goals. Individuals who participate on the TAP will work to ensure continuity and adherence to study timelines. A formal Manual of Procedures has been developed and the TAP will also reinforce existing data use and ownership agreements, and articulate processes for disseminating study findings. Importantly, if the falls prevention program proves successful, the tribe will enthusiastically attempt to sustain the program with support of the Senior Centre and the CHRs.

Aim 2 is to culturally-adapt the evidence-based OEP for use with the Zuni elder population. During the first 12 months, we will complete the program adaptation in three phases: (1) Provide education for CHRs to understand elder falls risks, the CDC STEADI toolkit, and OEP protocol training. The purpose is to provide CHRs with sufficient education to conduct focus groups and begin the adaption of OEP intervention and education (control arm) materials. The CHRs will convene 4 focus groups of 7–10 Zuni elders to provide feedback on the study protocol and materials. These resources will be culturally adapted to ensure possible phrasing and statements considered I:ba’naye (‘Taboo’ in Shiwi) are not used. Once the materials are adapted, the CHRs will conduct 1–2 focus groups to obtain feedback on the revised materials for the intervention (2) CHRs will work with project investigators to culturally adapt the STEADI participant resources (control arm) including home safety checklist and OEP participant manual (intervention arm) and; (3) once all relevant intervention content is revised, the CHRs will receive additional in depth training on the use of the measurement instruments, as well as the OEP protocol, materials and training/operations manual. Trained Zuni facilitators from our ongoing Zuni Health Initiative study will moderate the focus groups. Focus group discussions will be audio recorded and transcribed using NVivo.

The STEADI *Check Your Home for Safety* will be adapted in addition to the STEADI educational materials, the OEP participant manual will be adapted to (1) ensure that exercises are perceived as culturally appropriate; (2) instructions are at an appropriate literacy level; and (3) link exercise prescription to valued daily activities to improve perception of personal relevance to healthy aging [19].

Once all relevant intervention content is revised, the CHRs will receive additional in-depth training on the use of the measurement instruments, as well as the OEP protocol, materials and training/operations manual.

The education control group will receive education via the CDC *Stay Independent* checklist as part of the fall risk screening process. Additionally, the education control group will receive information from the CDC STEADI toolkit during the home visits. The education control group will review: *Stay Independent, What You Can Do to Prevent Falls, Check for Safety,* and *Postural Hypotension: What It Is and How to Manage It*. The CHRs will provide 10 min of falls prevention education per document and these resources will provide structure to CHR-delivered education and remain in people’s homes. The education control group will be assessed on the same schedule as the OEP intervention group.

All CHRs will be trained in the OEP intervention, the control group education protocol and the outcome measures. To ensure competency, four CHRs will be trained and supervised by a physiotherapist, who is experienced with implementing OEP in patient populations. The CHRs will receive comprehensive education on the use of the CDC STEADI toolkit for fall risk screening and the OEP adapted protocol. In addition, CHRs will review the online training developed by the University of North Carolina Geriatric Education Centre, “Preparing Community Health Workers and Promotors’ to Prevent and Reduce Falls Among Older Adults” [20]. The CHRs will achieve a score of 80% on a written and practicum exam for OEP, education control protocol, and home safety assessment protocol, and demonstrate competency during three simulated encounters, two initial participant encounters as well as quarterly observation of visits.

The CHRs will be trained on administration of all the research outcome measurements. The Timed Up and Go, the 30-Second Chair Stand Test, and the Four Stage Balance test are included in the STEADI toolkit, and have been widely used in the geriatric population [20–24]. Additional measures will include completing the monthly Falls and Exercise Diary, The Medical Outcomes Study Short Form 12 (SF-12v2) [25], two instruments from the Patient-Reported Outcomes Measurement Information System (PROMIS) [26, 27] Self-Efficacy for Managing Daily Activities – Short Form 4a and Ability to Participate in Social Roles and Activities- Short Form 4a. and Attitudes to Falls-related Interventions Scale (refs). The CHR will collect these measures at 3, 6, and 12 months. Through this training the Zuni CHRs will be trained and certified in falls prevention, leading strength and balance exercises (OEP) and data capture using REDCap™. In addition, the OEP training manual provides specific guidance on establishing a baseline and progression for the strengthening and balance exercises and walking program. This manual will serve as additional support and reinforcement of the original physical therapy-led OEP program.

Fidelity monitoring is detailed in the manual of operations and include protocols for study intake, screening, referral, intervention, home safety assessment, falls incidence and education control. Fidelity checklists will detail the essential elements of the intervention and the study measures. There will be 4 screenings observed, 4 simulated OEP sessions, and OEP visits quarterly in order to ensure program fidelity and participant safety. Delivery of the intervention will be supervised by DLW and JP. Teleconference technology is available on the Zuni pueblo and will be used to facilitate weekly meetings between the Zuni CHR staff, and UNM research staff during training and while the intervention is conducted.

Aim 3 is to compare the effectiveness of the adapted OEP to an education-based fall risk management program in improving strength and balance (decreasing falls risk) and reducing falls among Zuni Elders in a randomized controlled trial described below.

### Randomized controlled trial

#### Screening

Up to 400 elders will be screened for eligibility. If eligible, the CHRs will conduct the 3 gait, strength and balance tests and participants will complete a self-assessment for fall risk, Stay Independent, which the CHRs will review. Our pilot study used this self-assessment instrument and elders provided feedback that the questions were culturally appropriate and demonstrated willingness to provide responses [28].

#### Randomization

After the initial screening eligible and willing participants will be consented (n = 200). At that time, they will be randomly assigned to either OEP or the control education arm, stratifying within levels of fall risk (low, medium, or high) and employing cluster randomization by household family unit. Random assignment will be made according to a random allocation list that will be generated upon study initiation. This list will be generated such that sequentially consented participants will be assigned to study arms according to the falls risk of the first enrolled family member. Our intent is to obtain roughly equal numbers of participants within each of the strata defined by falls risk. To avoid contamination, we will apply a single intervention to the members of a particular household family unit. Therefore, it is possible that the number participants within a treatment group may not be perfectly balanced across falls risk strata. This will have minimal impact on the outcome of the trial, particularly relative to the contamination problems that it overcomes. Treatment groups will be assigned randomly to household family units grouped within blocks. Blocks will comprise 2 or 4 household family units, and the sequence of block sizes will be randomly generated. This dual random allocation will ensure that the study arms will remain relatively balanced while enhancing masking of treatment allocation to those enrolling study participants. The screenings will be conducted at the Zuni Health Initiative Site. We can also arrange for screening at the elder’s home if that is their preference.

#### The modified OEP intervention

We will implement a culturally modified protocol still aligned with the US translated OEP model [29]. The 6-month protocol involves 3 home visits during the first 4 weeks, then 2 visits during the second month, followed by monthly visits during months 3 to 6. The final visit will take place during the 12th month to perform the final assessment. The monthly visits will serve as a motivator as well as ensure safe performance of the home exercise program. Former OEP trials have determined that the monthly visits or phone calls improve compliance and motivation. Many Zuni elders do not have phones. We have enhanced the communication by assisting participants to obtain a federally funded Lifeline cell phone available to rural residents. This has been used successful in a previous trial by the VOS. The same home visit schedule will be applied to the education control group.

#### The OEP intervention first visit

The first visit is a 60–75-min session to provide individualized exercise instruction (flexibility, balance, and lower extremity strengthening), review and set a schedule for exercises and the walking program. Participants will be advised and provided written instructions to perform balance exercises a minimum of three times per week, strengthening exercises three times per week, and a walking program up to 30 min three times per week. We will use a comprehensive walking plan of the OEP program. They will be provided with a 12-month calendar to keep track of their exercises and falls reporting. This will be checked monthly by the CHRs. In brief, as part of our continuous training and in-service, CHRs will ask about typical walking activity and undergo training to identify individuals with unsafe walking patterns (very slow walking, unsteady walking, etc.). The CHRs will encourage participants to break up the 30-min walking sessions into 10-min increments. As participant’s confidence increases, the walking program can include outdoor walking if it is safe to do so. Participants who can walk independently (per inclusion criteria) are typically safe to walk in their homes. All walking programs will start in the home and give participants the option to extend their program outside of the home. Two transportation vehicles have been purchased by Zuni Pueblo as part of this trial. The Zuni Health Initiative Clinic will provide transportation for walking indoors if weather conditions do not permit outdoor walking.

#### Subsequent OEP visits

Subsequent visits will be 30–60 min and provide a review of exercises and ongoing instruction in safe performance and progression. This program will be individualized by the CHR with physical therapist consultation to ensure the safe progression of upper extremity support for balance and use of leg weights for leg strengthening exercises. The home exercise program will be modified to ensure plain language text at an appropriate literacy level and include an option for large print to accommodate vision difficulties. The CHRs will ensure completion of the Falls Diary and exercise calendars, which is included in the OEP implementation manual. Finally, CHRs will perform assessments as described during the third month, at the end of the intervention (6 months) and at the conclusion of the protocol (12 months) [24]. The intervention will be a 6-month intervention and 6-month follow up (total 12 months), which aligns with US translation of OEP (CDC Compendium), and contribute to the sustainability of the program, minimize dropouts and facilitate sufficient time for data analysis for all enrolled participants [18, 30].

#### Education control arm

One hundred participants will be randomized into an education control arm. As part of the screening, they will have completed the *Stay Independent* self-assessment for falls risk. Within two months of the screening, CHRs will visit the education control participants’ homes and implement the education intervention. The education control protocol involves review of the: *Stay Independent, What You Can Do to Prevent Falls, Check for Safety,* and *Postural Hypotension: What It Is and How to Manage It* documents. These resources will provide structure to CHR-delivered education. Also, the control group will be provided information on available community resources (i.e. Zuni Senior Centre) and encouraged to implement home safety modifications. We estimate that CHRs will spend 10 min per document as part of the education control intervention. Participants will be provided written instructions and encouraged to maintain a falls and exercise calendar. The control group will receive monthly follow up visits to encourage compliance with the falls and exercise calendar, and undergo 3 reassessment visits at 3, 6, and 12 months. If an elder report a health concern, transportation and referral to Indian Health Service will be provided.

Aim 4 is to compare the effectiveness of the adapted OEP to an education-based fall risk management program in improving overall health status, self-management of daily activities, and social engagement. To achieve this, we will administer the Medical Outcomes Study Short Form 12 SF-12 Health Survey and Short Form PROMIS measures Self-Efficacy for Managing Daily Activities, and Ability to Participate in Social Roles and Activities to all randomized participants in both groups, at baseline, 6 and 12 months.

The Medical Outcomes Study Short Form 12 (SF-12v2) measures an individual’s perspective on health-related quality of life [25]. It addresses 8 domains; physical functioning, role; physical, bodily pain, general health perceptions, vitality, social functioning, role-emotional, and mental health. However, due to the limited number of items, composite scores represent physical health and mental health. We have validated the SF-12v2 instrument in Zuni adults [31]. Prior research has shown that higher SF-12v2 scores are associated with healthier behaviours, improved quality of life, and reduction in emergency department visits [32]. In addition to the SF-12v2 instrument, two measures from the Patient-Reported Outcomes Measurement Information System (PROMIS) will be utilized. The PROMIS Item Bank v1.0—Self-Efficacy for Managing Daily Activities – Short Form 4a demonstrates one’s confidence in performing activities of daily living and instrumental activities of daily living; essential for aging in place and preserving independence [33]. The PROMIS Item Bank v2.0—Ability to Participate in Social Roles and Activities- Short Form 4a measures one’s perceptions of ability to engage in leisure activities, family and work activities, and social engagement [26]. Both instruments are 4-question surveys and will take less than 10 min to administer. Finally, CHRs will administer the Attitudes to Falls-Related Interventions Scale (AFRIS) to measure attitudes to the fall risk management intervention [34–36]. It identifies positive, negative, and neutral attitudes as well as change over time. List of study related measures collected at baseline, 6 months, and 12-month assessment is presented in Table 1.

**Table 1:** Schedule of testing

	Baseline	3 m	6 m	12 m
**Specific Aim 3 Measures**				
Brief Medical History & Comprehensive Falls Risk Screening	x			
STEADI Fall Risk Self-Assessment, Stay Independent	x			
STEADI Strength and Balance Tests (TUG, 30 Second Chair Stand, Four Stage Balance Test)	x	x	x	X
Monthly Falls Diary, Exercise Calendar and home visits	x	x	x	X
**Specific Aim 4 Measures**				
Attitudes to Falls-Related Interventions Scale (AFRIS)	x			X
Medical Outcomes Study Short Form 12 (SF-12v2)	x		x	X
PROMIS Self-Efficacy for Managing Daily Activities	x		x	X
PROMIS Ability to Participate in Social Roles and Activities	x		x	X
**Clinical Characteristics**				
Anthropological Measurement (Ht & Wt)	x			X
Orthostatic Blood Pressure	x	x	x	X

#### Data analysis plans

The analysis for Aim 2 will involve the audiotapes for each focus group being transcribed and thematically analysed using NVivo 10. The UNMCTSC will provide expertise in coding for qualitative data. Two trained coders will review the transcripts and propose codes based on the issues raised. A final codebook will be developed after several iterations of transcript review by the two coders and in consultation with the entire team. Zuni CHRs and team members will have critical input in the analysis and interpretation of the data. Data will also be verified by CHRs and the Tribal Advisory Panel. Once the team (VOS, CH, DLW, JP) have reached consensus on the coding template, the UNMCTSC will import the full set of transcripts into NVivo 10, qualitative data analysis software, for coding and analytical purposes. The analyses will inform and guide the process of culturally adapting OEP and education materials.

Adherence to OEP intervention is an ancillary outcome of Aim 2. Adherence will be measured by self-report and also assessed via STEADI strength and balance testing at 3, 6, 12 months. The emphasis is on strength and balance assessment and the maintained functional improvements to demonstrate continued adherence. Additional indicators of adherence will include OEP sessions attendance records, and completion of the monthly exercise and falls calendars. We have devised a simple two-point system for determining adherence. Participants will receive one point for each of two benchmarks achieved: (1) > 70% performance of prescribed individualized OEP sessions during the past 3 months; and (2) 100% of activity log books kept over the past two weeks and monthly falls calendars for 12 months. Data on “appointments kept” will include study-related appointments. Maintenance of a home exercise program is key to sustain the benefits of an exercise intervention. If functional measures using the STEADI strength and balance testing and performed at 3, 6, 12 months are maintained this will demonstrate continued adherence. We will follow intervention and control participants for 6 months post-intervention depending on time of initial enrolment and consideration of the 5-year timeline.

Changes in strength and balance (TUG, 30 Second Chair Stand, and Four Stage Balance test) are the primary outcome measures for this study. The CHRs will perform these assessments at baseline, 3, 6, and 12 months. Falls incidence is a secondary outcome and compared between the intervention and education groups. Fall incidence will be monitored prospectively monthly by falls calendars. We note that the high level of clinical surveillance of Zuni elders by the Zuni Senior Centre makes them an ideal population to record fall incidence and minimizes recall bias. Participants will be asked to maintain a monthly fall calendar throughout the investigation. A fall will be defined as an unexpected event where an individual unintentionally comes to the ground or lower level [37]. The CHRs will ask participants if they have fallen at every home visit. If a fall is reported, details of the fall(s), timing of fall, information about injury, and other pertinent information will be attained through standardized questionnaire. If an acute injurious fall is reported, CHRs will utilize a protocol (to be developed) for referral to IHS for follow up care and evaluation for ongoing participation in the trial. The fall diary will allow for the determination of number of falls, number of injurious falls and fall rate per person year. Fall diaries are the gold standard in fall assessment [38]. Collecting information on timing of fall will also allow for the examination of impact of the intervention on fall incidence.

The analysis plan for Aim 3, the randomized controlled trial, will employ an intention-to-treat analysis. We will first compare baseline demographic and clinical characteristics, such as age, sex, and fall risk score, between study groups using chi-square tests for categorical data and t-tests for continuous data. This will enable us to evaluate the degree to which the randomization scheme balanced these characteristics between the study groups, stratified by falls risk. As we expect randomization to provide for equivalent study groups at baseline, we do not expect to need to adjust for baseline characteristics in our primary analyses. However, we will change these plans should there be major differences in participant characteristics between treatment arms.

Falls incidence is not the primary outcome measure for this trial. Changes in balance and strength, as measured by the TUG, 30 Second Chair Stand, and Four Stage Balance tests will serve as primary outcome measures, which are important correlates of falls risk. We will employ linear mixed effects models to evaluate the significance of the treatment effect by testing for an interaction between treatment arm and time for these longitudinally measured assessments of the outcome measures. These models enable the efficient use of all measured data values while appropriately accounting for repeated measurements, and also the clustering that will be induced by stratified randomization of participants within household family units into strata defined by falls risk. Falls incidence is a secondary outcome. We will estimate and compare the incidence of falls observed within the two study groups over the study’s observation time using a score test from a Cox regression model that accounts for the clusters of individuals within household family units who were assigned to treatment groups within falls risk strata. In the absence of clustering, this is equivalent to a log rank test for time-to-event outcomes. This approach utilizes data from study participants, nested within household family units, while accounting for their time on study: those enrolled earlier in the study will have greater time in follow-up. We will also use Cox proportional hazards models to perform regression analyses that compare treatment groups while adjusting for potential differences in baseline characteristics.

In summary, first, we will follow the intent-to-treat principle in our primary analyses. Second, we will additionally adjust for the potential bias created by contamination using a contamination adjusted intention-to-treat analysis through the use of an instrumental variables (IVs) approach [39]. Third, we will carefully evaluate modelling assumptions (e.g. normality and variance structures in the mixed models, and proportionality of hazards in the survival models) in order to extract appropriate conclusions from the data. We will take corrective action when necessary, such as applying data transformations, to ensure that statistical modelling assumptions are met by the data. Finally, we will evaluate the degree to which missing data might have on study conclusions by performing sensitivity analyses that implement different rules from those induced by the intent-to-treat principle. Should these analyses suggest that the results differ by data missingness, we will explore the use of multiple imputation approaches and pattern mixture modelling approaches to determine the degree to which results differ under various missing data assumptions before making a final conclusion from the between-group comparisons.

In addition to these primary analyses, we will conduct a series of additional analyses to determine the degree to which the comparisons of interest might be influenced by patient characteristics. In particular, we are interested in whether the study comparisons are confounded, or modified by falls risk or complications of diabetes such as orthostatic hypertension/dizziness. We will perform analyses that examine the degree of confounding of the intervention effects by including them as covariates in the primary analysis models, and examine the degree to which they might modify intervention effects by adding their interactions with treatment arm to the primary analysis models.

#### Power and sample size

The primary outcomes of this study are changes in the TUG, 30 Second Chair Stand, and Four Stage Balance tests. These outcomes were selected because they are related to falls risk. Although the aim is to eventually demonstrate a reduction in falls incidence, the Zuni elder population is not big enough to provide adequate statistical power for this outcome. We do plan to extend the follow-up falls calendars to capture these outcomes, and this will provide critical information for future studies. The accepted degree of reduction in falls that is imparted by a traditional OEP is 32% [38, 39]. Because the percent reduction in falls does not directly correlate with a quantitative effect size that will be estimable from the data addressing our primary hypotheses, and because the outcome measures do not perfectly predict the probability of falls, it is likely that the detectable effect size that would be meaningful in the context of reducing falls would need to be a large one in order to suggest that the intervention is likely to have the desired effect on falls incidence. Therefore, the study was powered to detect at least moderate effect sizes. Specifically, we computed the number of participants to enrol into the study that would provide us with at least 80% power to detect an effect size of 0.67 for an improvement in the outcome measures over time between the treatment arms using a two-sided, 0.05 level test of between-group differences over time. Power estimates were obtained from linear mixed effects models that assumed within-subject correlations of 0.3, and within-cluster correlations of 0.1, and also corrected for 3 primary tests of significance. This computation suggested that data from 84 participants in each treatment arm would be required. In order to allow for the potential for dropouts, or for the possibility that the treatment might not be quite as efficacious as desired, 100 study participants per treatment arm will be enrolled. Even if 20% of the participants drop out of the study, this sample size will provide at least 80% power to detect effect sizes of 0.59 – nearly the target of 0.67, and still not a large effect size.

The analysis plan of Aim 4 will utilize linear mixed effects models to compare changes in outcome measurements between treatment arms, as outlined in the analysis plans for Specific Aim 2. These analyses will account for repeated measurements made over time for participants, when appropriate, and will be performed on a data scale that meets modelling assumptions. As in the first aim, an intent-to-treat paradigm will be followed, and sensitivity analyses to examine the impact of drop-out and missing data on the results of the comparisons between groups.

For the power and sample size of Aim 4 we selected the sample size in order to have adequate power for our primary outcomes in Specific Aim 2. With 100 participants per treatment arm, the trial will have 80% power at a two-sided 0.05 level of significance to detect effect sizes of 0.5 in magnitude for changes in quantitative measurement between treatments, even accounting for multiple testing and for potential attrition of up to 20% of the 200 seniors aged ≥ 65 to be enrolled in the trial.

## Discussion

### Spirit of partnership and community engagement

The randomized controlled trial study design comes from numerous conversations and significant compromise on the part of the tribe. In the past, a randomized controlled trial (RCT) design has not been agreed to by the Zuni Pueblo. However, feedback from unsuccessful grant applications clearly demonstrated that this study would not be funded without a RCT study design. We are grateful to the Zuni tribal council for co-designing the current design.

In accordance with the tribal council’s contribution to the study design, all study participants will receive a home safety inspection and offered modification for trip hazards, such as improved lighting (e.g. night lights) and bathtub mats according to guidelines in the STEADI resource Check Your Home for Safety at the beginning of enrolment into the intervention or control group. This is an ethical approach due to the high falls risk in those elder who are randomized to control group and this will be included in our bias assessment.

In the spirit of community engagement and the requirement of the request for proposals, the Zuni community will receive approximately 70% of grant funds over the five years of the study. Up to four Zuni CHR’s and one Zuni coordinator will receive salary support from the grant. Thus, all Zuni research positions will be held by residents of the Zuni Pueblo and all participants will be recruited from within Zuni Pueblo. This assures the Zuni community receives maximum benefits from the trial and continues to grow workforce capacity and skills within the Zuni Pueblo.

According to our existing agreement, all presentations and publications will be reviewed and approved by the Tribal leadership at least 30 days prior to presentation and presented to the Zuni tribe first. Once the trial is completed, we will address local and national dissemination of our results by leveraging resources from UNM’s nationally recognized Project ECHO (Extension for Community Health Outcomes) to make local teleconference presentations to professional and lay audiences. In addition, we will present our scientific results at national and international meetings (Geriatric Society of America, Center for Disease Control and Prevention, Indian Health Service, International Association of Gerontology and Geriatrics, and the New Zealand Association of Gerontology). The Albuquerque Area Southwest Tribal Epidemiology Center will lead the dissemination effort to disseminate findings to rural and urban fall prevention stakeholders along with support by the New Mexico Adult Falls Prevention Coalition and Albuquerque Area Tribal Injury Prevention Coalition. The Falls Coalition is a member of the National Council on Aging Falls Free Initiative, which distributes state fall prevention updates to a national fall’s stakeholder list serve comprised of all state falls coalitions in the US.

Sustainability will be two-fold. We will leverage healthcare insurance payment policies by facilitating Medicaid reimbursement for CHRs and strengthening Indian Health Service (IHS) partnerships to include CHR-delivered OEP into IHS service and referral system. Secondly, the Governor is willing to provide additional support as needed to sustain a successful home based CHR delivered OEP.

### Strengths and limitations

This trial has limitations and strengths. Limitations include that we will not employ a blind assessment, as this would require 4 additional in-person visits and would increase participant burden. Blind assessment is not feasible in this close-knit community. Rather, we will be vigilant in monitoring for assessment fidelity and control for bias by household family unit cluster randomization in the statistical analyses. Another potential limitation is that the OEP is designed for individuals to complete without any additional assistance. If a family member assists an individual in remembering to do the exercises, or how to do the exercises, this will not impact on outcomes. The CHRs will collect information about type and frequency of this type of assistance and this data will be used as a covariant in the analysis.

Strengths include that we brought together a team of qualified investigators who present a unique opportunity to increase understanding of effective approaches to fall risk reduction in an indigenous group with a high burden of fall-related injury. All the investigators have a history of working with indigenous communities both in the US and New Zealand. Another strength is proposing to expand the scope of practice for CHRs by adding fall risk screening and exercise instruction to their available services. The STEADI toolkit offers comprehensive, but simple methods to perform screenings and within the fall prevention research community, appropriate adult falls training content has been developed for CHRs, trialled and free to interested stakeholders. Nevertheless, it remains probable that real and meaningful lifestyle change is unlikely to occur in a sustainable way if individuals do not find internal motivation to modify fall risk factors and continue exercising after the 12-month protocol. The CHRs at Zuni have been shown to effectively implement other health promotion protocols including diabetes and kidney health management. We also conducted a focus group with the current long-term CHRs that demonstrated their enthusiasm for the protocol, a foundation of basic knowledge in exercise and participant safety, and essential cultural knowledge for successful implementation. The rationale behind such internal motivation is likely to vary from person to person, and only through the provision of a flexible, culturally sensitive, person-centered, and carefully considered fall risk management plan will the medical and public health community finally succeed in getting large proportions of elders with elevated fall risk to change their behaviour. Thus, this may prove to be this research protocol’s greatest strength.

#### Confidentiality

Participation in research will involve a loss of privacy, but information about subjects will be handled as confidentially as possible. Representatives of the University of New Mexico Health Sciences Center Human Research Review Committee that oversees human subject research, the Food and Drug Administration or other regulatory agencies will be permitted access to subjects’ records. Also, subject’s participation in the study and information in their study records may be disclosed to their doctors and nurses, and may be disclosed as otherwise provided by law. However, subjects name will not be used in any published reports about this study. Hard copy of all study results will be kept in locked cabinets. Electronic copies of study results will be kept in pass word protected files in computers located in offices of study personnel. Only authorized study personnel, representatives of the NIH and the Human Research Review Committees of the UNMHSC and I H S will have access to study results. The study operates under an NIH Certificate of Confidentiality, which helps researchers protect the privacy of participants in research studies. This certificate means that researchers cannot be forced to tell people who are not connected to this study, including courts, about your participation unless you give written consent. Conducting the study under this Certificate helps to ensure your privacy. In addition, if publications or presentations results from this research, your name or any other personal identifier will not be used.

## Data Availability

The unique research resources established and created in our efforts to develop newly validated methods for fall risk assessment and management / interventions, along with any new Native American specific educational material in Zuni that evolve from the studies, will be available to the scientific community. However, we will follow institutional, tribal, IHS and local IRB guidelines, as well as proprietary mandates. Biological samples are not collected and/or archived for any future studies. We are also committed to sharing other methodological resources with NIH-sponsored projects and coordinating with NIH-supported data coordinating canters as requested. We plan to data readily available to the research community once the experimental and analytical tasks on the studies are complete. We will coordinate data sharing with the NIH and Zuni tribal leadership and make available both the raw data and analysed data upon request from the PIs (Shah and Herman).

## Abbreviations

AFRIS: Attitudes to Falls- Related Interventions Scale
AI: American Indian
CHR: Community Health Representative
CDC: Center for Disease Control and Prevention
CITI: Collaborative Institutional Training Initiative
ECHO: Extension for Community Health Outcomes
IHS: Indian Health Service
OEP: Otago Exercise Programme
PROMIS: Patient-Reported Outcomes Measurement Information System
REDCap: Research Electronic Data Capture
RCT: Randomized Controlled Trial
SF-12v2: Medical Outcomes Study Short Form 12
STEADI: Stopping Elderly Accident Deaths and Injuries
TUG: Timed up and Go
TAP: Tribal advisory panel
UNM: University of New Mexico
UNMCTSC: University of New Mexico Clinical and Translational Science Centre

## References

[1] Dixon M, Roubideaux Y (2001). Promises to keep: public health policy for American Indians and Alaska Natives in the 21st Century.

[2] Murphy T, Pokhrel P, Worthington A, Billie H, Sewell M, Bill N (2014). Unintentional injury mortality among American Indians and Alaska Natives in the United States, 1990 2009. Am J Public Health.

[3] New Mexico's indicator-based information system (NM-IBIS): health indicator report of injury - unintentional injury deaths, 2018 -https://ibis.health.state.nm.us/indicator/view/InjuryUnintenDeath.InjCause.html. Accessed 2/1/2022.

[4] Albuquerque area southwest tribal epidemiology center (AASTEC), 2016. AASTEC Tribal BRFSS Project 2007–2014. Retrieved from https://www.aastec.net/wpcontent/uploads/2016/09/AASTEC_factsheet_injuryprevention_2016_final_nocropmarks.pdf. Accessed 2/1/2022.

[5] New Mexico Department of Health. Unintentional fall-related injuries among older adults in New Mexico, 2014. Retrieved from http://healthinsight.org/images/locations/nm/PDFs/Falls/Falls_Report_2014_Final.pdf. Accessed 2/1/2022.

[6] Tinetti ME (2003). Clinical practice Preventing falls in elderly persons. N Engl J Med.

[7] Close JC (2005). Prevention of falls in older people. Disabil Rehabil.

[8] Chang JT, Morton SC, Rubenstein LZ, Mojica WA, Maglione M, Suttorp MJ, Roth EA, Shekelle PG (2004). Interventions for the prevention of falls in older adults: systematic review and meta-analysis of randomised clinical trials. BMJ.

[9] Berry SD, Miller RR (2008). Falls: epidemiology, pathophysiology, and relationship to fracture. Curr Osteoporos Rep.

[10] Rubenstein LZ, Josephson KR (2006). Falls and their prevention in elderly people: what does the evidence show?. Med Clin North Am.

[11] Kelsey JL, Procter-Gray E, Hannan MT, Li W (2012). Heterogeneity of falls among older adults: implications for public health prevention. Am J Public Health.

[12] Goins RT, Innes K, Dong L (2012). Lower body functioning prevalence and correlates in older American Indians in a Southeastern Tribe: the native elder care study. JAGS.

[13] Campbell AJ, Robertson MC, Gardner MM, Norton RN, Tilyard MW, Buchner DM (1997). Randomised controlled trial of a general practice programme of home based exercise to prevent falls in elderly women. BMJ.

[14] Campbell AJ, Robertson MC, Gardner MM, Norton RN, Buchner DM (1999). Falls prevention over 2 years: a randomized controlled trial in women 80 years and older. Age Ageing.

[15] Campbell AJ, Robertson MC. Otago exercise programme to prevent falls in older adults. http://www.hfwcny.org/Tools/broadcaster/Upload/Project13/Docs/Otago_Exercise_Programme.pdf. Retrieved on 14 Mar 2019.

[16] Campbell, A.J. and Robertson, M.C. Otago exercise programme to prevent falls in older adults. http://www.hfwcny.org/Tools/BroadCaster/Upload/Project13/Docs/Otago_Exercise_Programme.pdf. Retrieved on 14 Mar 2019.

[17] Robertson MC, Campbell AJ, Gardner MM, Devlin N (2002). Preventing injuries in older people by preventing falls: a meta-analysis of individual-level data. J Am Geriatr Soc.

[18] Stevens JA, Burns ER. A CDC Compendium of effective fall interventions: what works for community-dwelling older adults. 3rd edn. Atlanta: Centers for Disease Control and Prevention, National Center for Injury Prevention and Control, 2015.

[19] National center for injury prevention, 2015. tools to implement otago exercise program: a program to reduce falls, 1^st^ ed. Retrieved from https://www.med.unc.edu/aging/cgec/exercise-program/tools-for-practice/ImplementationGuideforPT.pdf. Accessed 2/2/2022.

[20] UNC Center for Aging & Health, Carolina Geriatric Education Center, National Community Health Worker Training Center. n.d. Preparing Community Health Workers and Promotores to prevent and reduce falls among older adults. Retrieved from http://www.aheconnect.com/cgec/cdetail.asp?courseid=cgec6. Accessed 2/2/2022.

[21] Jones C, Rikli R (1999). A 30-s chair-stand test as a measure of lower body strength in community-residing older adults. Res Q Exerc Sport.

[22] Podsiadlo D, Richardson S (1991). The timed "Up & Go": a test of basic functional mobility for frail elderly persons. J Am Geriatr Soc.

[23] Shumway-Cook A, Brauer S (2000). Predicting the probability for falls in community-dwelling older adults using the Timed Up & Go Test. Phys Ther.

[24] Rossiter-Fornoff J, Walf S, Wolfson L (1995). A cross-sectional validation study of the FICSIT common data base static balance measures. Gerontol A Biol Sci Med Sci.

[25] Gandek B, Ware JE, Aaronson NK, Apolone G, Bjorner JB, Brazier JE, Kassa S, Lepleg A, Prieto L, Sullivan M (1998). Cross-validation of item selection and scoring for the SF-12 Health Survey in nine countries: results from the IQOLA Project. J Clin Epidemiol.

[26] Hong I, Velozo CA, Li CY, Romero S, Gruber-Baldini AL, Shulman LM (2016). Assessment of the psychometrics of a PROMIS item bank: self-efficacy for managing daily activities. Qual Life Res.

[27] Hahn EA, DeWalt DA, Bode R, Garcia SF, DeVellis RF, Correia H, Cella D, PROMIS Cooperative Group (2014). New English and Spanish social health measures will facilitate evaluating health determinants. Health Psychol.

[28] Popp J, Waters DL, Leekity K, Ghahate D, Bobelu J, Tsikewa R (2017). Using the centers for disease control and prevention’s stay independent checklist to engage a community of American Indians and raise awareness about risk of falls, 2016. Prev Chronic Dis.

[29] Shubert TE, Smith ML, Goto L, Jiang L, Ory MG (2017). Otago exercise program in the United States: comparison of two implementation models. Phys Ther.

[30] National Council on Aging. Otago exercise program guidance statement, 2017. Retrieved from https://www.ncoa.org/wp-content/uploads/2017-OEP-Guidance-Statement.pdf. Accessed 2/2/2022.

[31] Cukor D, Cohen L, Cope EL, Ghahramani N, Hedayati SS, Hynes DH, Shah VO (2016). Patient and other stakeholder engagement in patient-centered outcomes research institute funded studies of patients with kidney diseases. Clin J Am Soc Nephrol.

[32] Shah VO, Carroll C, Mals R, Ghahate D, Bobelu J, Sandy P (2015). A home-based educational intervention improves patient activation measures and diabetes health indicators among Zuni Indians. PLOS1.

[33] Courtney M, Edwards H, Chang A, Parker A, Finlayson K, Hamilton K (2009). Fewer emergency readmissions and better quality of life for older adults at risk of hospital readmission: a randomized controlled trial to determine the effectiveness of a 24-week exercise and telephone follow-up program. J Am Geriatr Soc.

[34] Hahn EA, DeWalt DA, Bode R, Garcia SF, DeVellis RF, Correia H, Cella D (2014). PROMIS Cooperative Group New English and Spanish social health measures will facilitate evaluating health determinants. Health Psychol.

[35] Yardley L, Donovan-Hall M, Francis K, Todd C (2006). Older people's views of advice about falls prevention: a qualitative study. Health Educ Res.

[36] Yardley L, Donovan-Hall M, Francis K, Todd C (2007). Attitudes and beliefs that predict older people's intention to undertake strength and balance training. J Gerontol B Psychol Sci Soc Sci.

[37] Lord SR. Falls in older people : risk factors and strategies for prevention. 2nd ed. Cambridge, New York: Cambridge University Press; 2007. pp. xi, 395 p.

[38] Lamb SE, JorstadStein EC, Hauer KK, Becker C (2005). Development of a common outcome data set for fall injury prevention trials The prevention of falls network Europe consensus. J Am Geriatr Soc.

[39] Sussman JB, Hayward RA (2010). An IV for the RCT: using instrumental variables to adjust for treatment contamination in randomised controlled trials. BMJ.

